# Novel ‘schizophrenic’ diblock copolymer synthesized via RAFT polymerization: poly(2-succinyloxyethyl methacrylate)-*b*-poly[(*N*-4-vinylbenzyl),*N*,*N*-diethylamine]

**DOI:** 10.1080/15685551.2016.1239165

**Published:** 2016-10-21

**Authors:** Aliyeh Ghamkhari, Bakhshali Massoumi, Mehdi Jaymand

**Affiliations:** ^a^ Department of Chemistry, Payame Noor University, Tehran, Iran; ^b^ Research Center for Pharmaceutical Nanotechnology, Tabriz University of Medical Sciences, Tabriz, Iran

**Keywords:** Poly(2-succinyloxyethyl methacrylate), poly[(*N*-4-vinylbenzyl),*N*,*N*-diethylamine], RAFT, diblock copolymer, self-assembly, ‘schizophrenic’

## Abstract

This article describes the synthesis and characterization of a novel ‘schizophrenic’ diblock copolymer [poly(2-succinyloxyethyl methacrylate)-*b*-poly[(*N*-4-vinylbenzyl),*N*,*N*-diethylamine)]; PSEMA-*b*-PVEA] via reversible addition of fragmentation chain transfer (RAFT) polymerization technique. The chemical structures of all samples as representatives were characterized by means of Fourier transform infrared (FTIR), and ^1^H nuclear magnetic resonance (NMR) spectroscopies. The molecular weights of PHEMA and PVEA segments were calculated to be 9770 and 12,630 gmol^−1^, respectively, from ^1^H NMR spectroscopy. The self-assembly behavior of the synthesized PSEMA-*b*-PVEA diblock copolymer was investigated by means of ^1^H NMR spectroscopy, dynamic light scattering (DLS) measurements, and transmission electron microscopy (TEM) observation. The average sizes of the PSEMA-*b*-PVEA micelles at pHs 3.0, 6.0, and 10.0 were obtained to be 294, 237, and 201 nm, respectively, from DLS analysis. The zeta potential measurements at various pHs demonstrated that the synthesized PSEMA-*b*-PVEA diblock copolymer has zwitterionic properties, and the range of isoelectric point’s (IEP’s) was determined as 5.8–7.3. It is expected that the synthesized PSEMA-*b*-PVEA diblock copolymer considered as a prospective candidate in nanomedicine applications such as drug delivery, mainly due to its excellent ‘schizophrenic’ micellization behavior.

## 1. Introduction

Since the first reports of ‘schizophrenic’ micellization by Armes and coworkers in 1998, diblock copolymers with precisely defined structures and ‘schizophrenic’ micellization behavior have been stimulated a great deal of research effort based on their potential applications in nanomedicine (e.g. controlled release of guest molecules, diagnostics, tissue engineering, and biosensors).[1–6] The ‘schizophrenic’ micellization behavior is originated from changes in hydrophilicity of the segments of a block copolymer, as some environmental parameters such as temperature, pH, and ion strength are finely adjusted.[7–11]

Among the various stimuli, the pH is the best and first choice, mainly due to its simplicity in needing to just introduce some acid or base, as well as water solubility and great potential in biomedical applications such as drug or gene release.[12–14] It is important to note that, physiological pH changes from 1 to 7.5 depend on the different locations in the body. Thus, ionizable polymers with a pK_a_ value in the range of 3–10 are potential candidates for pH-responsive systems. In this respect, the polymeric compounds possesses weak acids and bases groups such as phosphoric acid, carboxylic acids, and amines exhibit a change in the ionization state upon variation of the pH.[5,15–18]

In general, various methods have been explored to synthesize diblock copolymers, such as coupling reaction (e.g. ‘click’ chemistry),[19,20] ‘living’ anionic and cationic polymerizations,[21,22] ring-opening polymerization (ROP),[23,24] and reversible deactivation radical polymerization (RDRP; otherwise known as controlled or ‘living’ radical polymerization) approaches.[25–31] Based on RDRP technique, three main types of controlled radical polymerizations have been investigated as follows: nitroxide-mediated polymerization (NMP),[32,33] atom transfer radical polymerization (ATRP),[34,35] and reversible addition of fragmentation chain transfer (RAFT) polymerization.[36,37] Among these approaches, the RAFT polymerization can be conducted in more moderate conditions and operated conveniently. In addition, there are no metal contaminants in the resultant polymer. To date there have been numerous reports detailing the successful synthesis of diblock copolymers through RAFT polymerization. For example, using the RAFT polymerization Su et al. [38], described the synthesis of a block copolymer of poly[2-(2-methoxyethoxy)ethyl methacrylate]-*block*-poly[*N*-(4-vinylbenzyl)-*N*,*N*-diethylamine] (PMEO_2_MA-*b*-PVEA) with tunable lower critical solution temperature (LCST), and the upper critical solution temperature (UCST) in the alcohol/water mixture.

In this contribution, the synthesis, characterization, and self-assembly behavior of a novel diblock copolymer, poly(2-succinyloxyethyl methacrylate)-*b*-poly[(*N*-4-vinylbenzyl),*N*,*N*-diethylamine] (PSEMA-*b*-PVEA) is reported. For this purpose, a RAFT agent (4-cyano-4-[(phenylcarbothioyl)sulfanyl]pentanoic acid), and VEA monomer were synthesized. Afterward, HEMA and VEA monomers was copolymerized via RAFT polymerization technique to afford PHEMA-*b*-PVEA diblock copolymer. Finally, the PSEMA-*b*-PVEA was synthesized by the esterification of PHEMA-*b*-PVEA diblock copolymer through the reaction between hydroxyl groups of the PHEMA block with an excess amount of succinic anhydride.

## 2. Experimental

### 2.1. Materials

2-Hydroxyethyl methacrylate, and 4-chloromethyl styrene (Merck, Darmstadt, Germany) were dried over calcium hydride, vacuum-distilled, and then stored at −20 °C prior to use. The initiator 2,2′-azobisisobutyronitrile (AIBN, Fluka, Switzerland) was recrystallized from ethanol at 50 °C before use. Tetrahydrofuran (THF, Merck) was dried by refluxing over sodium, and distilled under argon atmosphere before use. Diethyl amine, 4,4′-azobis(4-cyanopentanoic acid), bromobenzene, and pyridine were purchased from Sigma-Aldrich (USA), and were used as received. All other reagents were purchased from Merck and purified according to the standard methods.

### 2.2. Synthesis of RAFT agent

**Scheme 1.**
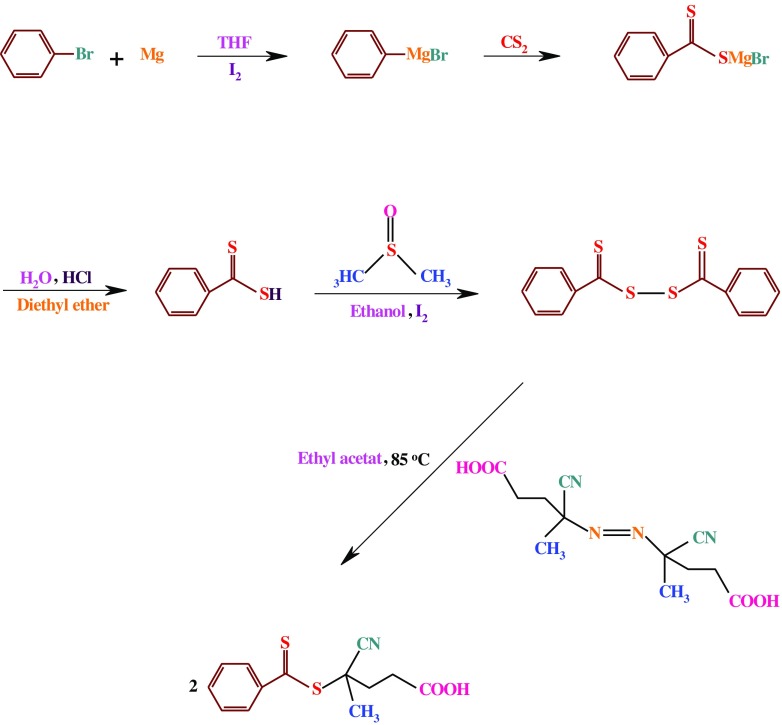
Synthesis route of the RAFT agent.

The RAFT agent, 4-cyano-4-[(phenylcarbothioyl)sulfanyl]pentanoic acid, was synthesized according to the literature (Scheme 1).[39]

### 2.3. Synthesis of *N*-(4-vinylbenzyl)-*N*,*N*-diethylamine (VEA) monomer

**Scheme 2.**
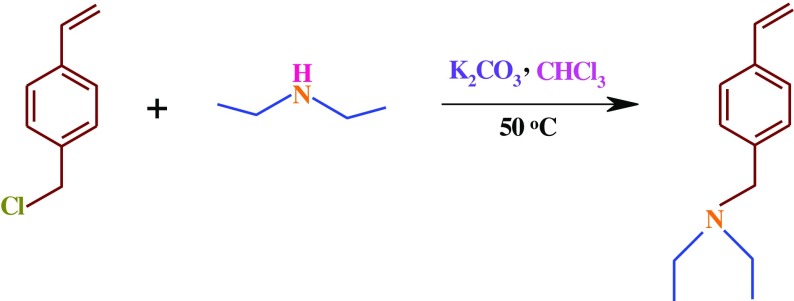
Synthesis route of the VEA monomer.

A 250 mL three-neck round-bottom flask equipped with a condenser, septum, gas inlet/outlet, and a magnetic stirrer was charged with chloroform (150 mL), and diethyl amine (2.06 mL, 20 mmol). This solution was de-aerated by bubbling highly pure argon for 15 min, and then 2.76 g (20 mmol) of anhydrous potassium carbonate (K_2_CO_3_) was added to the flask. The reaction mixture was stirred for about 1 h at room temperature under argon atmosphere. At the end of this period, 4.2 mL (30 mmol) of 4-chloromethyl styrene was introduced with a syringe through the septum. The reaction mixture was stirred for about 24 h at 50 ± 3 °C under an argon atmosphere. The crude product was filtered, washed with water, the organic phase was dried using MgSO_4_, and then the solvent was removed by a rotary evaporator. Finally, the crud product was purified with silica-gel column chromatography using petroleum ether as the eluent to afford a colorless liquid (Scheme 2).

### 2.4. Synthesis of PHEMA via RAFT polymerization

**Scheme 3.**
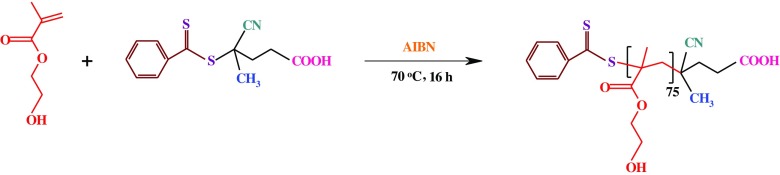
Synthesis of PHEMA via RAFT polymerization.

In a typical experiment, HEMA monomer (7.5 mL, 60.0 mmol), AIBN (7 mg, 0.04 mmol), and the synthesized RAFT agent (56.0 mg, 0.2 mmol) were dissolved in dried *N*,*N*-dimethylformamide (DMF; 10 mL). The reaction mixture was transferred to a polymerization ampoule, degassed with several freeze–pump–thaw cycles, sealed off under vacuum, and placed in an oil bath at 70 ± 3 °C for about 16 h. During the reaction, the viscosity of the mixture was observed to gradually increase. At the end of this time, the ampoule was cooled in ice/water bath, in order to quenching the polymerization. The mixture was diluted with DMF (10 mL), and precipitated in cold diethyl ether (200 mL). The product was dried under vacuum at room temperature (Scheme 3).

### 2.5. Synthesis of PHEMA-*b*-PVEA diblock copolymer via RAFT polymerization

The PHEMA-*b*-PVEA diblock copolymer was synthesized via RAFT copolymerization of VEA with employing the synthesized PHEMA as the macro-RAFT agent, and AIBN as the initiator. In a typical experiment, the macro-RAFT agent (PHEMA, 1.0 g, 0.09 mmol), AIBN (3.3 mg, 0.02 mmol), and VEA monomer (1.65 g, 8.7 mmol) were dissolved in 10 mL mixture of DMF/1,4-dioxane (4:1). The reaction mixture was transferred to polymerization ampoule, degassed with several freeze–pump–thaw cycles, sealed off under vacuum, and placed in an oil bath at 70 ± 3 °C for about 24 h. At the end of this time, the ampoule was cooled in ice/water bath, in order to quenching the reaction. The mixture was diluted with DMF (10 mL), and then precipitated in water/methanol mixture (50/50 (v/v), 200 mL). The product was filtrated, and dried in vacuum at room temperature to afford brown powder.

### 2.6. Synthesis of PSEMA-*b*-PVEA

**Scheme 4.**
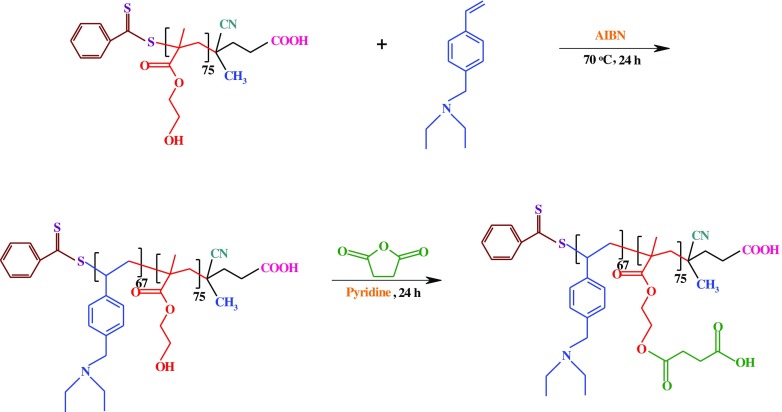
Synthesis route of PHEMA-*b*-PVEA, and PSEMA-*b*-PVEA.

The PSEMA-*b*-PVEA was synthesized by the esterification of PHEMA-*b*-PVEA diblock copolymer through the reaction between hydroxyl groups of the PHEMA block with an excess amount of succinic anhydride as follows. In a 100 mL round-bottom flask the PHEMA-*b*-PVEA diblock copolymer (1.0 g, 0.04 mmol) was dissolved in anhydrous pyridine (30 mL) at room temperature under argon flow. The succinic anhydride (0.50 g, 5 mmol) was added, and the reaction was allowed to proceed at room temperature for about 48 h. The reaction mixture was precipitated into 100 mL of methanol to remove the excess succinic anhydride. During precipitation some of the succinic anhydride was converted into monomethyl succinate, which was not removed. Thus, the precipitate was re-dissolved in 10 mL of pyridine and precipitated into 100 mL of diethyl ether, which is a better solvent for the monomethyl succinate byproduct (Scheme 4).

### 2.7. Characterization

Fourier transform infrared (FTIR) spectra of the samples were recorded on a Shimadzu 8101M FTIR (Shimadzu, Kyoto, Japan) in the range of wavenumbers from 4000 to 400 cm^−1^ with a resolution of 4 cm^−1^. For sample preparation the dry powders were grounded with potassium bromide (KBr), and compressing the mixture into disks. The proton nuclear magnetic resonance (NMR) spectra of the samples were recorded at 25 °C using an FT-NMR (400 MHz) Bruker spectrometer (Bruker, Ettlingen, Germany). The sample for NMR spectroscopy was prepared by dissolving about 10 mg of sample in 1 mL of deuterated solvent. Ultraviolet-visible (UV-vis) spectra were recorded with a Shimadzu 1650 PC UV-vis spectrophotometer (Shimadzu, Kyoto, Japan) in the wavelength range of 600–400 nm. Dynamic light scattering (DLS) measurements were performed by Nanotrac Wave™ (Microtrac, San Diego, CA, USA) at room temperature. Samples were prepared as 0.5% (w/v) solutions in distilled deionized water, with the solution pH being adjusted by adding HCl or NaOH where appropriate. The solutions were stirred for 3 h and then left unstirred overnight. The samples were then ultrafiltered through a 0.20 μm nylon membranes (Micron Separations, Westboro, MA, USA) before being analyzed. Transmission electron microscopy (TEM) images of the synthesized PSEMA-*b*-PVEA diblock copolymer at various pHs were taken on a Philips CM10-TH microscope (Phillips, Eindhoven, The Netherlands) with a 100 kV accelerating voltage. The samples were prepared by placing a drop of the PSEMA-*b*-PVEA solution on a carbon-coated copper grid.

## 3. Results and discussion

As a decisive fact, emergence of nanotechnology has provided exciting new possibilities for development of novel systems for application in various multidisciplinary fields such as nanomedicine. Stimuli-responsive block copolymers with self-assembly behavior are one of the advances and innovations results in the field of nanotechnology. These types of polymeric materials received a great deal of attention in the past decade, mainly due to their unique physicochemical properties and potential applications in pharmaceutics, rheology modifiers, colloidal stabilization, coatings, and templates for the preparation of nanomaterials.[3,40] As mentioned previously, the objective of study is to synthesis and characterization of a novel ‘schizophrenic’ diblock copolymer (PSEMA-*b*-PVEA) via RAFT polymerization technique.

### 3.1. Characterization of RAFT agent, and VEA monomer

**Figure 1.**
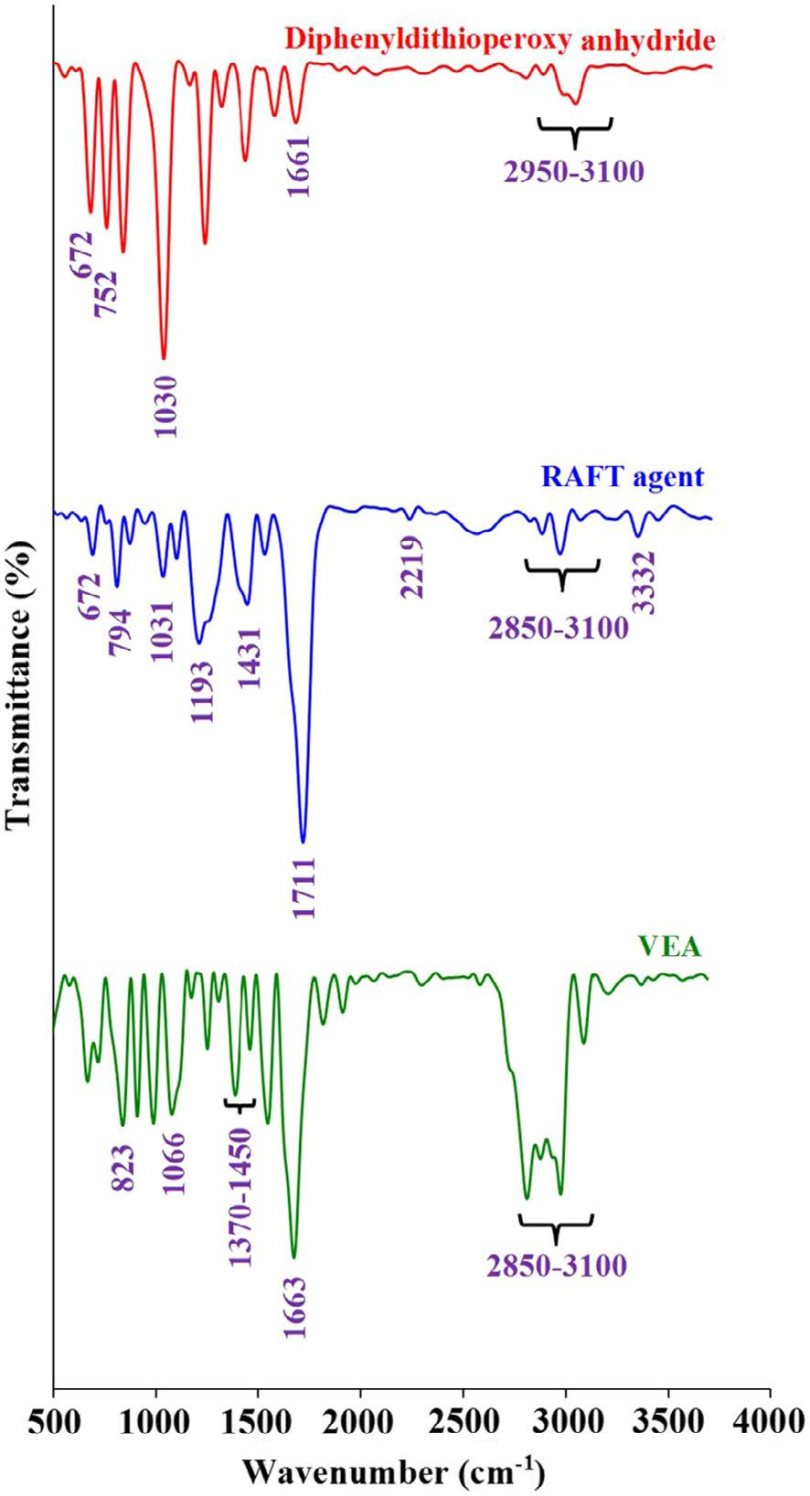
The FTIR spectra of diphenyldithioperoxy anhydride, RAFT agent, and VEA monomer.

The FTIR spectra of diphenyldithioperoxy anhydride, RAFT agent, and VEA monomer are shown in Figure 1. The FTIR spectrum of diphenyldithioperoxy anhydride exhibits characteristic absorption bands due to the stretching vibrations of aromatic C–H at 3100–2950 cm^−1^ region, γ(C–H) in the aromatic ring at 752 cm^−1^, the stretching vibrations of aromatic C=C at 1661 and 1429 cm^−1^, the C–S stretching vibration at 672, and the stretching vibration of C=S group at 1031 cm^−1^. In contrast, the FTIR spectrum of the RAFT agent shows the stretching vibrations of aromatic and aliphatic C–H at 3100–2850 cm^−1^ region, γ(C–H) in the aromatic ring at 794 cm^−1^, cyanide stretching vibration at 2219 cm^−1^, carbonyl stretching vibration at 1711 cm^−1^, and the C–H bending vibration at 1431 cm^−1^. In addition, the stretching vibrations of C=S and C–S groups are appeared at 1031 and 672 cm^−1^, respectively.

The FTIR spectrum of the VEA monomer exhibits the stretching vibrations of aromatic and aliphatic C–H at 3100–2850 cm^−1^ region, γ(C–H) in the aromatic ring at 823 cm^−1^, the stretching vibrations of vinyl and aromatic C=C as a strong band at 1663 cm^−1^, C–N stretching vibration at 1066 cm^−1^, the C–H bending vibrations at 1450–1370 cm^−1^, and weak aromatic overtone and combination bands in the 2200–1700 cm^−1^ region. These FTIR spectra verify the successful synthesis of the RAFT agent and VEA monomer.

**Figure 2.**
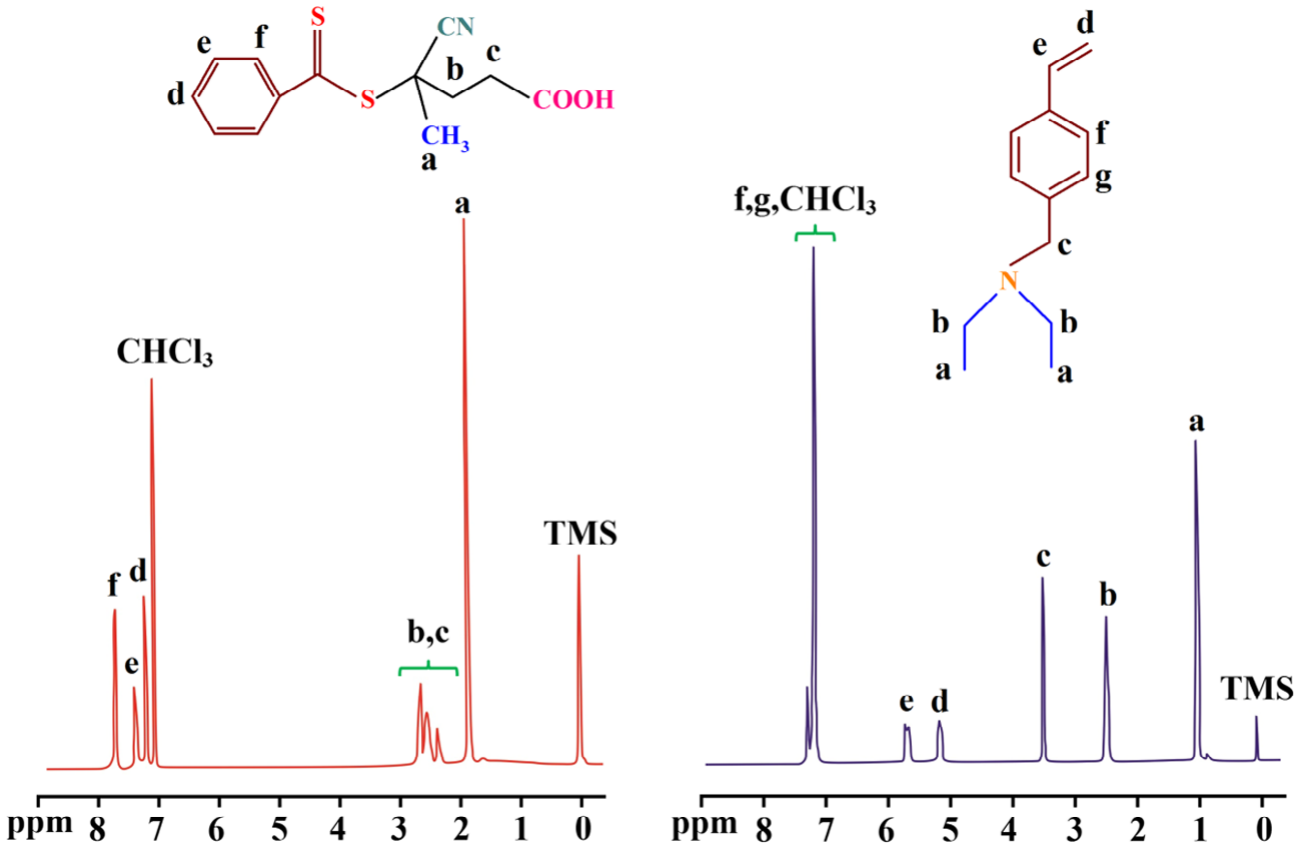
The ^1^H NMR spectra of the synthesized RAFT agent and VEA monomer (CDCl_3_, 25 °C).

The synthesized RAFT agent and VEA monomer were further characterized by means of ^1^H NMR spectroscopy as shown in Figure 2. The ^1^H NMR spectrum of the RAFT agent shows the chemical shifts at 1.95 and 2.40–2.90 ppm related to the methyl and methylen protons, respectively. The aromatic protons of the phenyl ring are appeared at 7.30–8.00 ppm.

In ^1^H NMR spectrum of the VEA monomer the chemical shifts at 0.9–1.20, and 2.40–2.70 ppm are related to the methyl and methylen protons of the VEA, respectively. The chemical shift at 3.60 ppm is assigned to the N–CH_2_ protons, and the chemical shifts at 5.20 and 5.80 ppm are related to the vinyl protons of the VEA monomer. In addition, the aromatic protons of the VEA are appeared at 7.20–7.50 ppm.

**Figure 3.**
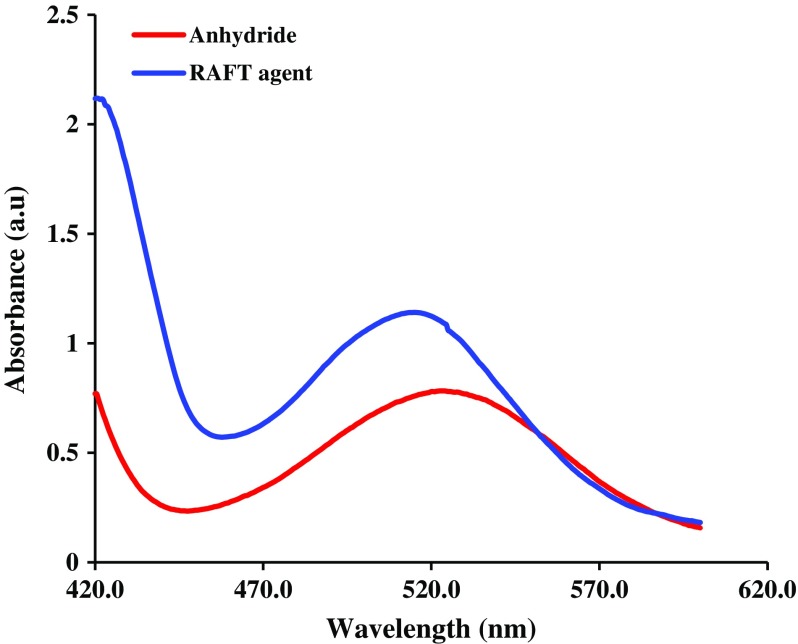
Absorbance spectra of diphenyldithioperoxy anhydride, and RAFT agent in dichloromethane solution.

Furthermore, the successful synthesis of diphenyldithioperoxy anhydride, and RAFT agent was verified by UV-vis spectroscopy. As seen in Figure 3, the UV-vis spectra of both samples were characterized by one electronic transition between 600 and 400 nm. The UV-vis spectrum of the diphenyldithioperoxy anhydride shows maximum absorption peak at ~525 nm attributable to *n*–π* transition. In contrast, the UV-vis spectrum of the synthesized RAFT agent exhibits maximum absorption peak at ~514 nm attributable to *n*–π* transition. In the case of RAFT agent the electron-withdrawing groups (cyanide and carboxyl) cased the blue shift in comparison with diphenyldithioperoxy anhydride sample.

### 3.2. Characterization of PSEMA-*b*-PVEA diblock copolymer

**Figure 4.**
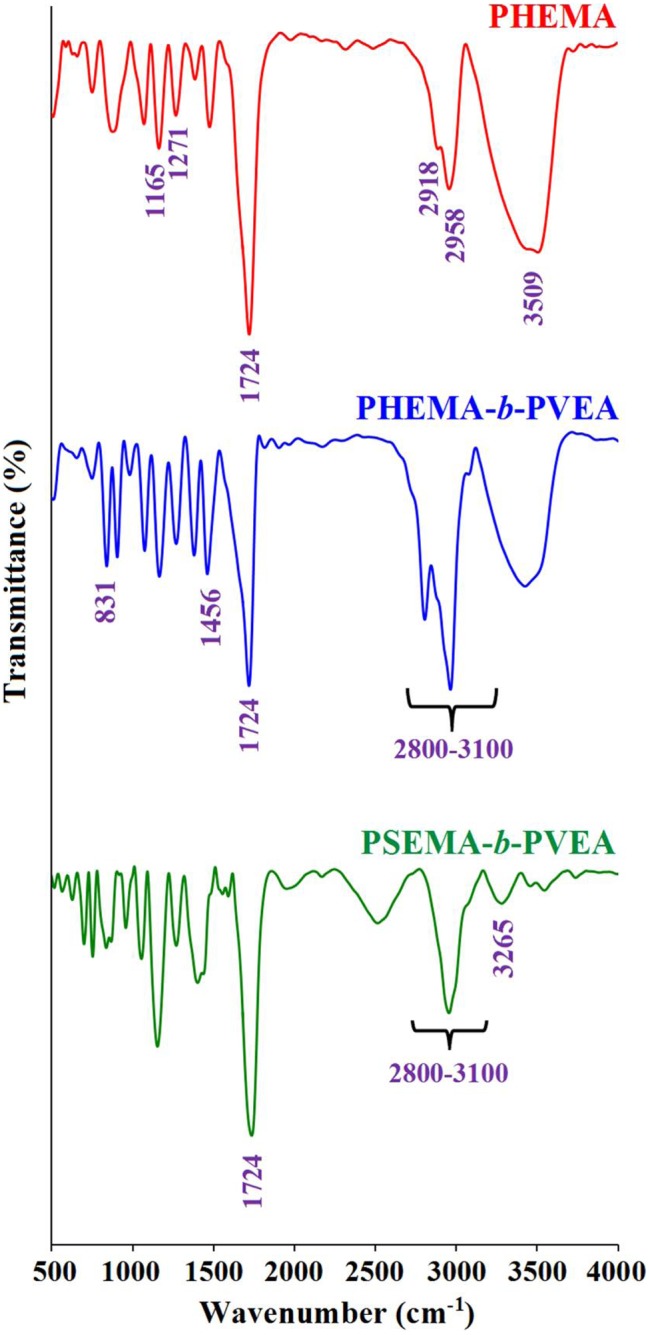
The FTIR spectra of the PHEMA, PHEMA-*b*-PVEA, and PSEMA-*b*-PVEA.

The FTIR spectra of the PHEMA, PHEMA-*b*-PVEA, and PSEMA-*b*-PVEA samples are shown in Figure 4. The FTIR spectrum of the PHEMA shows the characteristic absorption bands due to the stretching vibration of carbonyl group at 1724 cm^−1^, aliphatic C–H stretching vibrations at 2918 and 2958 cm^−1^, C–H bending vibration at 1466 cm^−1^, stretching vibration of C–O group at 1373 cm^−1^, and C–O–C stretching vibration at 1271 cm^−1^. The broad strong band centered at 3509 cm^−1^ is related to the hydroxyl group of the PHEMA. The FTIR spectrum of the PHEMA-*b*-PVEA diblock coplolymer shows the typical bands corresponding to the both PHEMA and PVEA segments. The main absorption bands in this sample could be listed as: aliphatic and aromatic C–H stretching vibrations at 3100–2800 cm^−1^ region, aromatic C=C stretching vibration at 1456 cm^−1^, γ(C–H) in the aromatic ring at 831 cm^−1^, and hydroxyl stretching vibration as a broad strong band centered at 3509 cm^−1^.

After esterification of PHEMA-*b*-PVEA diblock copolymer with succinic anhydride the most significant changes in the FTIR spectrum are the disappearance of hydroxyl stretching vibration at 3509 cm^−1^, and appearance of new band at 3265 cm^−1^, which is related to hydroxyl group of the carboxylic acid.

**Figure 5.**
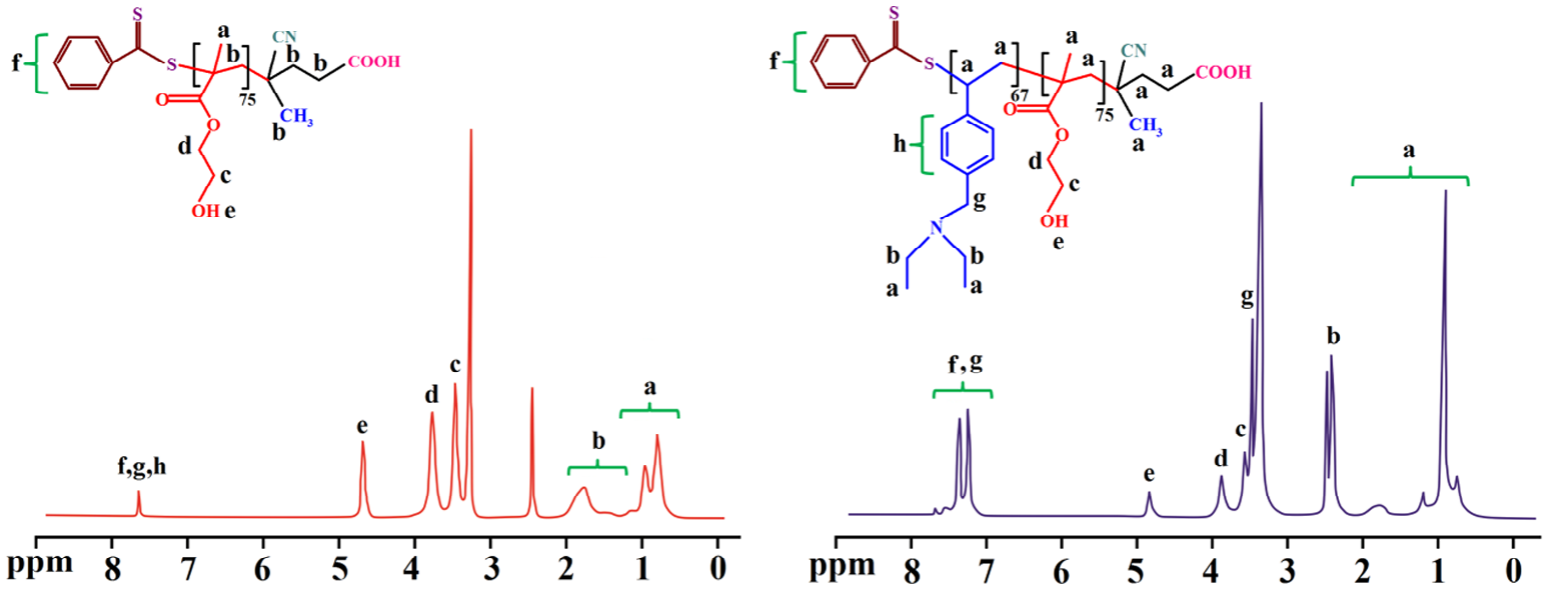
The ^1^H NMR spectra of the PHEMA and PHEMA-*b*-PVEA (DMSO-*d*_6_, 25 °C).

The synthesized PHEMA and PHEMA-*b*-PVEA diblock copolymer were further characterized by means of ^1^H NMR spectroscopy (Figure 5). In ^1^H NMR spectrum of the PHEMA the chemical shifts at 7.95 is related to aromatic protons of RAFT agent. The methyl and methylene protons of the PHEMA backbone are appeared at 0.75–0.95 and 1.75–2.05 ppm, respectively. The protons of the –CH_2_OH, and –OCH_2_ groups are observed at 3.55 and 3.85 ppm, respectively. In addition, the chemical shift at 4.80 ppm is related to the hydroxyl groups of the PHEMA.

**Figure 6.**
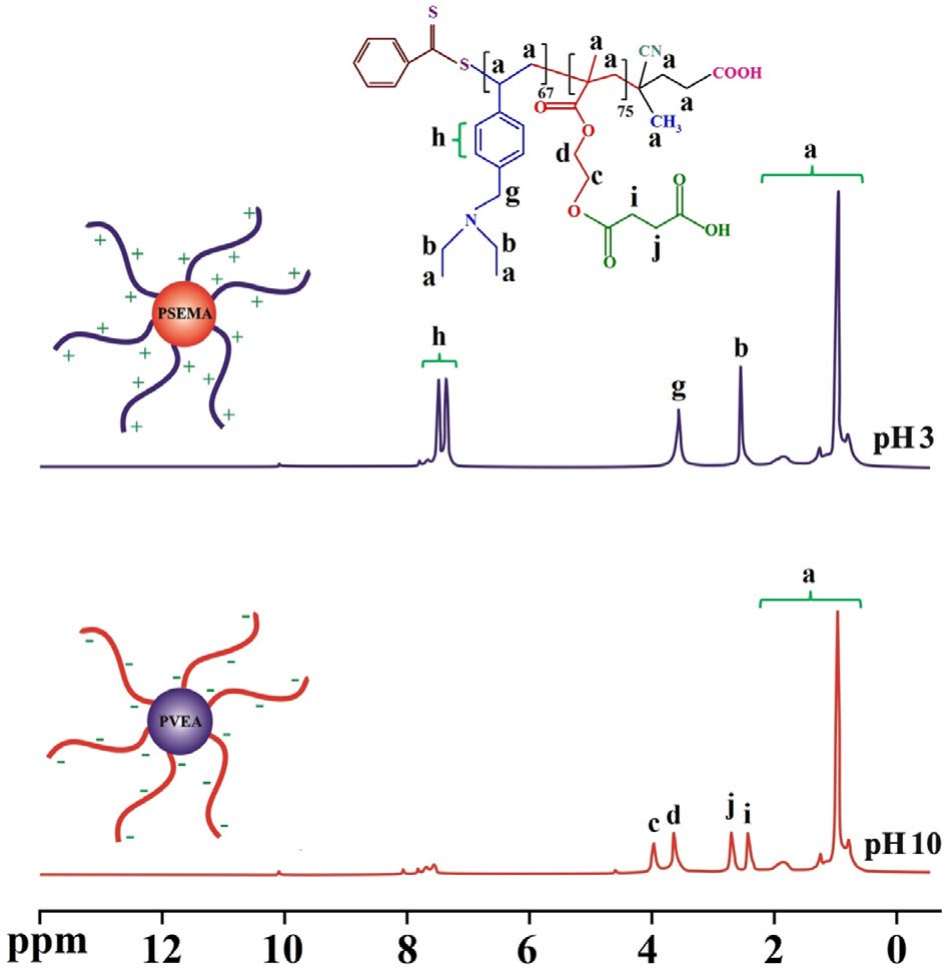
The ^1^H NMR spectra (D_2_O, 25 °C) for the PSEMA-*b*-PVEA ‘schizophrenic’ diblock copolymer recorded at pHs 3.0 and 10.0 using DCl and NaOD where appropriate.

**Figure 7.**
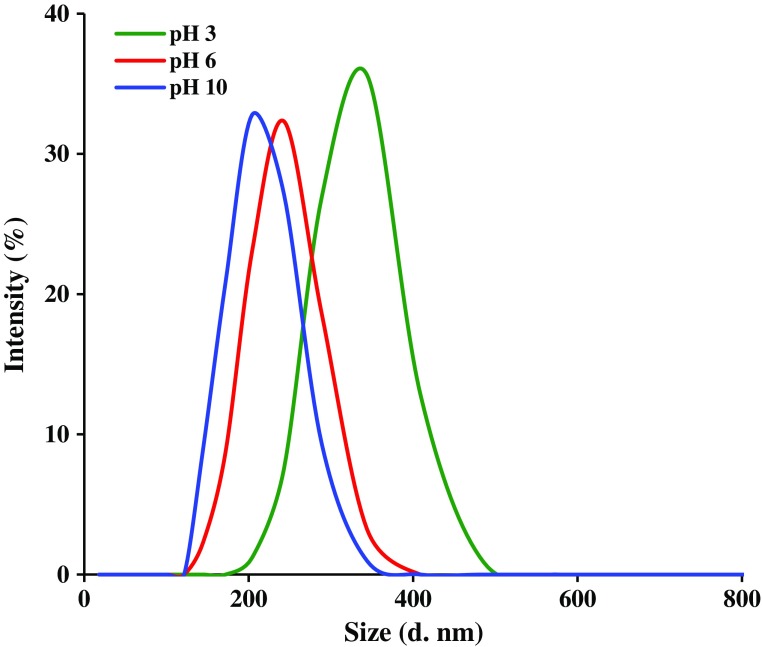
The DLS diagrams of the PSEMA-*b*-PVEA diblock copolymer at various pH values.

**Figure 8.**
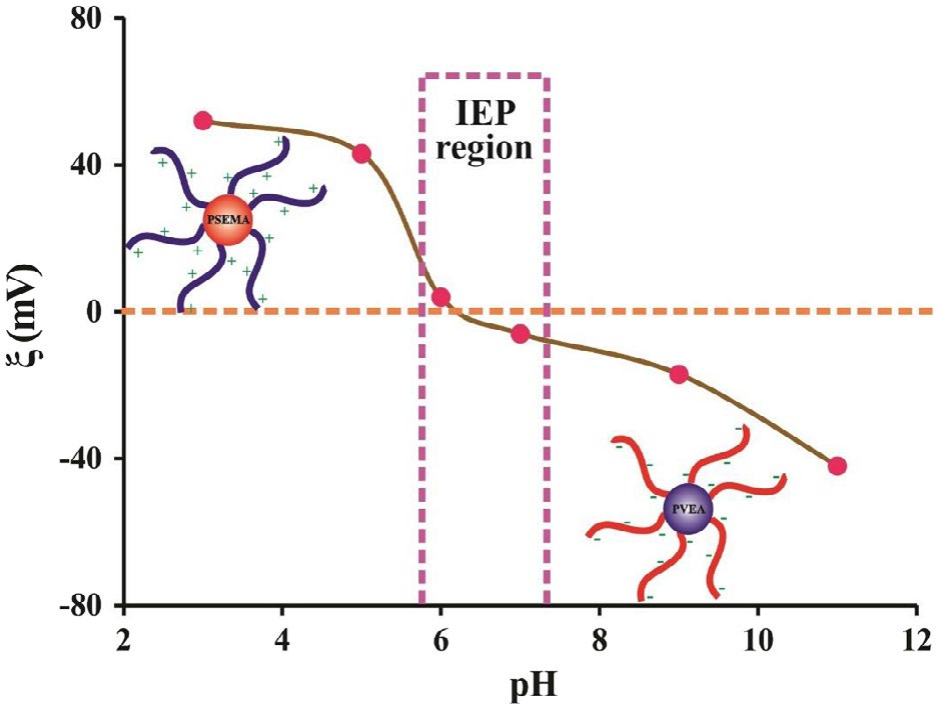
Variation of *ξ* potential with pH for a 0.50% (w/v) aqueous solution of the PSEMA-*b*-PVEA diblock copolymer.

**Figure 9.**
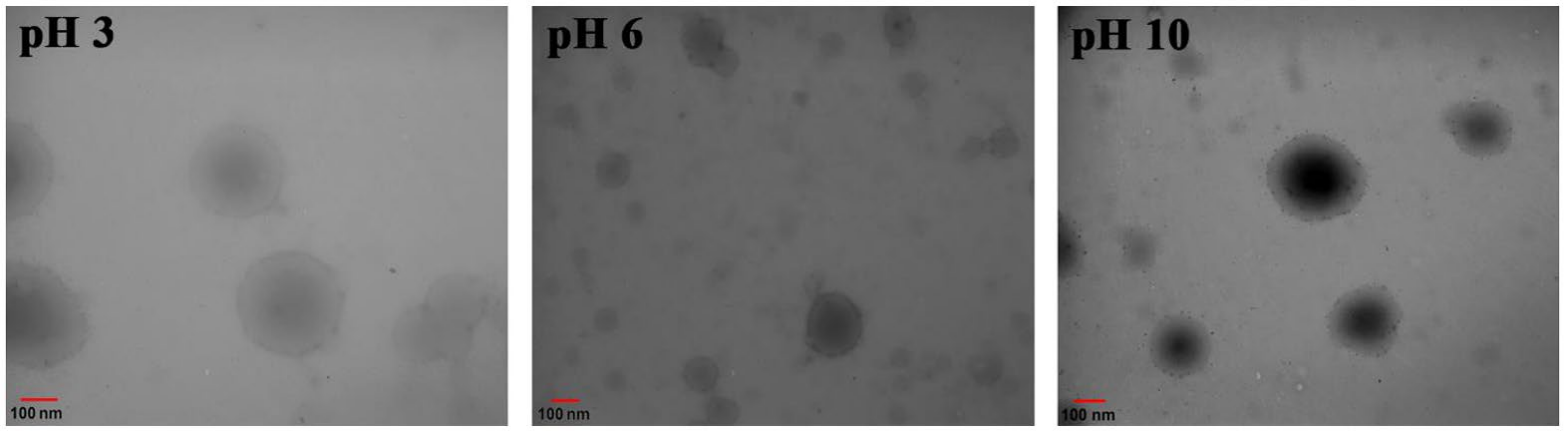
TEM micrographs of PSEMA-*b*-PVEA micelles at various pHs.

**Figure 10.**
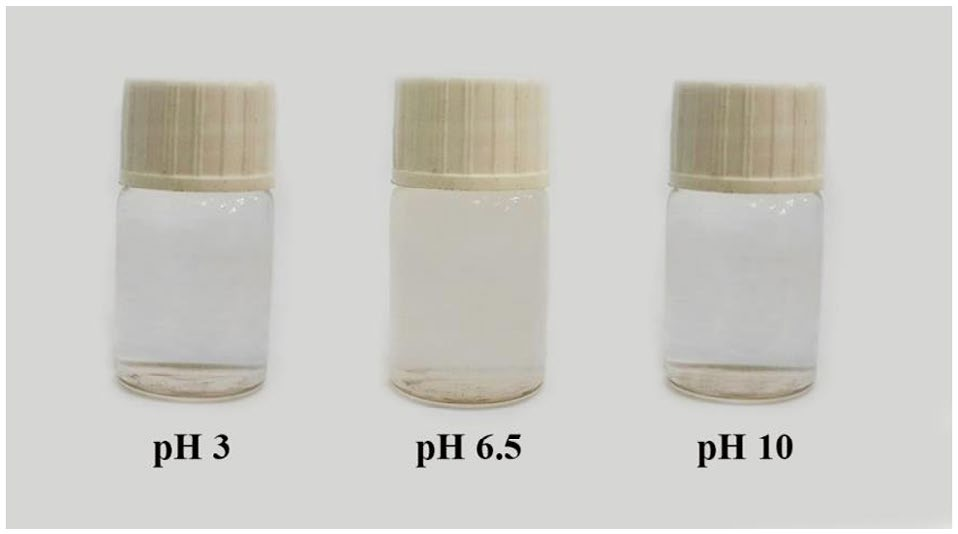
Digital photographs of PSEMA-*b*-PVEA diblock copolymer solution at pHs 3, 6.5, and 10.

The successful synthesis of the PHEMA-*b*-PVEA diblock copolymer is verified by the appearance of new chemical shifts at 7.20–7.60 and 3.50 ppm, which are related to the aromatic protons, and N–CH_2_ protons of the PVEA block. All other chemical shifts are labeled on ^1^H NMR spectrum of the PHEMA-*b*-PVEA diblock copolymer.

In the past few decades, ^1^H NMR spectroscopic analysis has been established as a powerful tool for the determination of some parameters of polymer such as composition, degree of polymerization (DP_*n*_), and number average molecular weight (*M*_*n*_).[41,42] The DP_*n*_ and *M*_*n*_ of the synthesized PHEMA and PHEMA-*b*-PVEA can be calculated from the ^1^H NMR data through the following equations.

$${\text{DP}}_{n,\text{HEMA}}=\;\frac{5\times I_{\left({\text{CH}}_{2}\text{OH}\right)}}{2\times I_{\left(\text{Aro-RAFT}\right)}}=75$$

$$M_{n,\text{PHEMA}}=\;\left[{\text{DP}}_{n,\text{HEMA}}\;\times M_{\text{HEMA}}\right]+\;M_{\text{RAFT}\;}=9766+279=10045$$

$$M_{n,\text{(PHEMA - b - PVEA)}}=\;M_{n,\text{Macro - RAFT}}+\left[\;\frac{(I_{\left({\text{N - CH}}_{\text{2}}\right)}\;\times 189.1)/4}{(I_{\left({\;\text{- CH}}_{\text{2}}\text{OH}\right)}\;\times 130.1)/2}\;\times \;M_{n,\text{Macro - RAFT}}\right]=22673$$

$$M_{n,\text{PVEA}}=12628$$

$${\text{DP}}_{n,\text{VEA}}=67$$

where *I*_i_ indicates integral intensity of protons i in the ^1^H NMR spectrum.

### 3.3. Investigation of self-assembly behavior

**Scheme 5.**
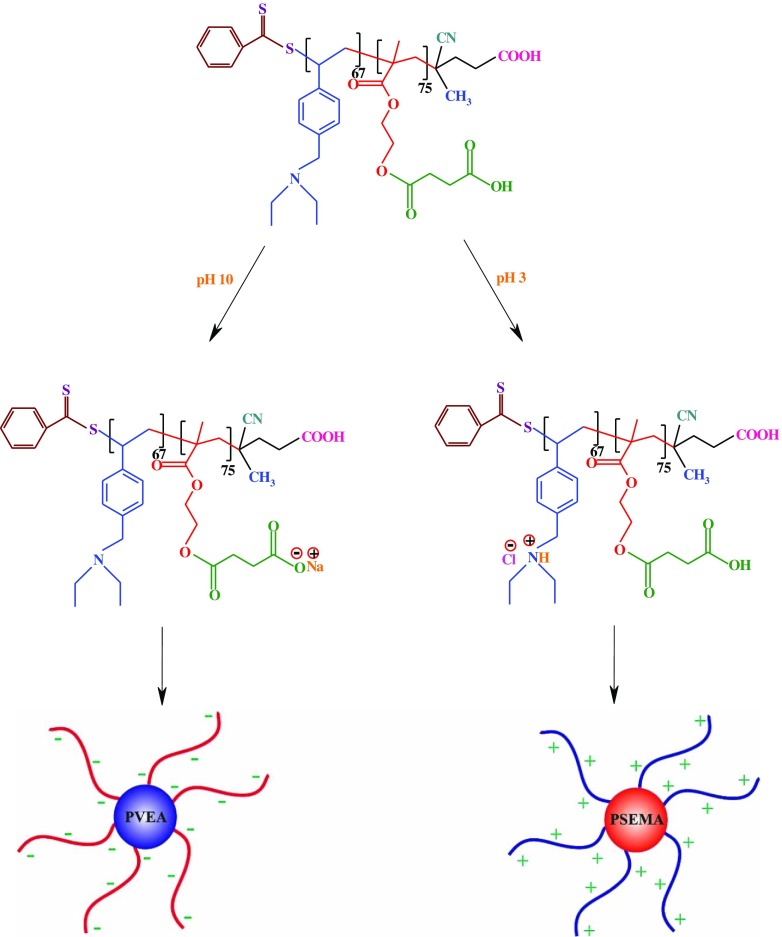
The possible schematic structure of PSEMA-*b*-PVEA micelles at pHs 10.0 and 3.0.

It is a decisive fact that block copolymers can self-assemble into two or more types of micelles in selective solvents with invertible structures under a combination of environmental stimuli (e.g. pH and temperature) or nanostructured thin films in the solid state.[43,44] The possible schematic structure of the PSEMA-*b*-PVEA micelles at pHs 10 and 3 are shown in Scheme 5.

The self-assembly behavior of the synthesized PSEMA-*b*-PVEA diblock copolymer was investigated by means of ^1^H NMR spectroscopy, DLS measurements, and TEM. The ^1^H NMR spectra of the PSEMA-*b*-PVEA diblock copolymer at pHs 10 and 3 are shown in Figure 6. As seen in ^1^H NMR spectrum of the PSEMA-*b*-PVEA diblock copolymer when the solution pH was adjusted to 10 with NaOD the chemical shifts related to the PVEA segment [b (2.45 ppm), g (3.5 ppm), and h (7.20–7.60 ppm)] were disappeared. In contrast, when the solution pH was adjusted to 3 with DCl the chemical shifts related to the PSEMA segment [i (2.40), j (2.65), d (3.55), and c (3.85)] were disappeared. On the other hand, this ^1^H NMR spectrum confirms that the PVEA block becomes protonated in acidic solution, so these aggregates should have cationic coronas. In conclusion, these ^1^H NMR spectra were demonstrated the ‘schizophrenic’ micellization of the synthesized PSEMA-*b*-PVEA diblock copolymer by pH stimuli.

Additional evidence on the self-assembly behavior of the PSEMA-*b*-PVEA diblock copolymer was obtained from DLS measurements. As seen in Figure 7, the micelle sizes decreases with an increase in solution pH. The average size of PSEMA-*b*-PVEA micelles at pHs 3.0, 6.0, and 10 were obtained to be 294, 237, and 201 nm, respectively. This is consistent with a higher degree of protonation of the PVEA vesicle coronas in water at lower pH, which leads to a stronger repulsive interaction and hence a bigger size.[45] In addition, polydispersity index (PDI) of the micelles at pHs 3.0, 6.0, and 10 were obtained to be 0.35, 0.58, and 0.31, respectively.

The self-assembly behavior of the PSEMA-*b*-PVEA diblock copolymer was further investigated by means of zeta potential (*ξ*) measurements at various pHs for a 0.50% (w/v) aqueous solution of the diblock copolymer as shown in Figure 8. Zeta potentials of the PSEMA-*b*-PVEA diblock copolymer at pHs 3.0, 5.0, 6.0, 7.0, 9.0, and 11.0 were measured to be +52, +43, +4, −6, −17, and −41, respectively. As seen in this figure, the zeta potentials decreased with increasing of solution pH, and at around pH 6 the net charge on the micelles were close to zero. Thus, we could conclude that the range of IEP’s for the synthesized PSEMA-*b*-PVEA diblock copolymer is in the pHs range of 5.8–7.3. On the other hand, the synthesized PSEMA-*b*-PVEA diblock copolymer can be defend as a zwitterionic diblock copolymer.[46]

At the end of this section, it should be pointed out that the PVEA is a new thermo-responsive polymer and its LCST was dependent on the polymer molecular weight and polymer concentration.[47,48] However, in the case of this study due to zwitterionic behavior, the synthesized PSEMA-*b*-PVEA diblock copolymer did not showed thermo-sensitivity.[46]

TEM images of the PSEMA-*b*-PVEA micelles at various pHs exhibited the presence of presumably spherical micelles of ~250–300, ~100–250, and ~100–200 nm in diameter at pHs 3, 6, and 10, respectively (Figure 9). It should be pointed out that the sizes estimated from TEM were systematically smaller than those obtained by DLS. This phenomenon can be described as follows.

(a) The TEM observation exhibits the dried aggregates or the core of the micelles, while DLS measurement detects the solvated micelles.
(b) The DLS analysis shows an intensity-weighted diameter, while the TEM observation exhibits a number-average diameter, and the former is always oversized than the latter.[7,49]

In addition, digital photographs of PSEMA-*b*-PVEA diblock copolymer solution at pHs 3, 6.5, and 10 are shown in Figure 10. In accordance to zeta potential measurement, around pH 6.5 the net charge on the micelles were close to zero and the solution became cloudy due to precipitation.

## 4. Conclusion

In summary, synthesis of a novel ‘schizophrenic’ diblock copolymer (PSEMA-*b*-PVEA) via RAFT polymerization technique was demonstrated. The molecular weights of PHEMA and PVEA segments were found to be 9770 and 12,630 gmol^−1^, respectively from ^1^H NMR spectroscopy. The self-assembly behavior of the synthesized diblock copolymer at various pH values were investigated by means of ^1^H NMR spectroscopy, DLS measurements, and TEM observation. The average sizes of the PSEMA-*b*-PVEA micelles at pHs 3.0, 6.0, and 10.0 were obtained to be 294, 237, and 201 nm, respectively from DLS measurements. The zeta potential measurements at various pHs exhibited that the PSEMA-*b*-PVEA diblock copolymer has zwitterionic properties, and the range of IEP’s was determined as 5.8–7.3. According to the results, we envision that the synthesized PSEMA-*b*-PVEA diblock copolymer can be considered as a prospective candidate in nanomedicine applications such as drug delivery, mainly due to its unique physicochemical properties. In conclusion, further experiments are under progress in order to evaluate the influence of the monomer compositions, and chains length on the physicochemical properties of the synthesized diblock copolymer.

## Disclosure statement

No potential conflict of interest was reported by the authors.

## Funding

This work was supported by Payame Noor University, and Research Center for Pharmaceutical Nanotechnology; Tabriz University of Medical Sciences.
